# Association between prognostic nutritional index and all-cause mortality and cardiovascular disease mortality in American adults with non-alcoholic fatty liver disease

**DOI:** 10.3389/fnut.2025.1526801

**Published:** 2025-02-10

**Authors:** Yuqing Lei, Shaohong Tao, Yubo Yang, Fang Xie, Weining Xie

**Affiliations:** ^1^Affiliated Guangdong Hospital of Integrated Traditional Chinese and Western Medicine of Guangzhou University of Chinese Medicine, Guangzhou University of Chinese Medicine, Foshan, Guangdong, China; ^2^Nanhai Hospital of Traditional Chinese Medicine, Jinan University, Foshan, Guangdong, China; ^3^Department of Liver Disease, Jinling Hospital Affiliated to Medical College of Nanjing University, Nanjing, Jiangsu, China; ^4^Guangdong Provincial Hospital of Integrated Traditional Chinese and Western Medicine, Foshan, Guangdong, China

**Keywords:** prognostic nutritional index, NAFLD, all-cause mortality, CVD mortality, non-linear, predictor, NHANES

## Abstract

**Background:**

The current research was to investigate the relationship between prognostic nutritional index (PNI) and mortality, with a focus on all-cause and cardiovascular disease (CVD) mortality, for those with non-alcoholic fatty liver disease (NAFLD).

**Methods:**

Data from 20,142 patients who participated in the National Health and Nutrition Examination Survey (NHANES), which was carried out between 2005 and 2014, were included in this research. To examine the relationship between PNI and both all-cause and cardiovascular mortality, we employed weighted Cox regression models with multiple variables. Kaplan–Meier survival curves were utilized to visualize the survival distribution across different levels of PNI. The non-linear association between PNI and mortality was addressed through penalized spline smoothing. Subgroup analyses were conducted to examine the potential influence of relevant clinical variables on the relationship between PNI and mortality. The precision of PNI in forecasting the outcome of survival was assessed as well using time-dependent receiver operating characteristic curve (ROC) analysis.

**Results:**

Kaplan–Meier analysis linked higher PNI to significantly reduced all-cause and CVD mortality. Multivariable Cox models demonstrated that increasing PNI consistently lowered mortality risks. With a threshold value of 50.5, the link between PNI and mortality showed a non-linear pattern after adjusting for confounding factors. Subgroup analyses confirmed robust associations, particularly in race, education, BMI, and fibrosis. Time-dependent ROC analysis highlighted the strong predictive performance of PNI across various time points.

**Conclusion:**

PNI played a significant role as an effective predictor of prognosis in individuals diagnosed with NAFLD.

## Introduction

Non-alcoholic fatty liver disease (NAFLD), which is unrelated to alcohol consumption, is an abnormal buildup of fat in the liver (1). It has emerged as one of the most prevalent liver diseases worldwide, affecting approximately 25% of the global population, with its prevalence rising rapidly in both developed and developing nations, especially in the US, impacting about one-third of the population (2). The disease spectrum spans from mild fat accumulation (steatosis) to more severe forms such as cirrhosis and liver cancer, often accompanied by systemic inflammation and insulin resistance (3). The rising global prevalence of NAFLD, especially its association with cardiovascular disease (CVD) and extrahepatic malignancies, has made it an important public health problem (4, 5). Advanced fibrosis remains a key prognostic indicator of liver-related mortality, while the pathophysiology of NAFLD is driven by insulin resistance, dysregulated lipid metabolism, and chronic inflammation, causing simple hepatic fat accumulation to non-alcoholic steatohepatitis, fibrosis, cirrhosis, and ultimately liver cancer (6). An increasing body of evidence suggests that NAFLD is associated with metabolic syndrome (MetS) and its related risks, including hypertension, obesity, type 2 diabetes, dyslipidemia, and cardiovascular disease, which in turn increases the likelihood of CVD and overall mortality (7). Emerging evidence has demonstrated that metabolic markers, including HDL-c, BMI, GGT, ALT, TB, DBIL, and TG, are significantly associated with the risk of NAFLD development (8). These markers have shown predictive value not only for obesity-related NAFLD but also for non-obese NAFLD (9). Moreover, studies have reported a strong association between NAFLD and hypertension. Elevated plasma aldosterone levels observed in hypertensive patients have been linked to an increased risk of incident NAFLD (10). Given the growing prevalence and the widespread health implications of NAFLD, particularly its association with metabolic and cardiovascular diseases, this condition presents a critical challenge for public health worldwide.

There is a clear relationship between NAFLD and nutrition, as dietary habits significantly influence the onset and progression of the disease (11). Studies have shown that diets high in saturated fats, trans fats, simple sugars, and animal proteins are detrimental to liver health, promote lipid accumulation, and contribute to metabolic disorders in NAFLD (11–13). The Prognostic Nutritional Index (PNI) is commonly used to assess an individual’s immune nutritional status and has been associated with outcomes across various diseases. PNI has been found to be used to predict mortality and cardiovascular risk, especially in populations with metabolic conditions like diabetes (14), obesity (15), or metabolic syndrome (16). In addition, higher PNI scores have been linked to a lower likelihood of developing complications such as diabetic kidney disease (DKD) (17), chronic kidney disease (CKD) (18), postoperative complications in hip fracture patients (19), and so on. Although several studies have explored the relationship between various nutritional markers and the progression of NAFLD, there is a lack of research specifically examining the role of PNI in predicting mortality outcomes in NAFLD patients. Furthermore, existing studies are often limited by small sample sizes or narrow population representation, leading to uncertainties about the generalizability of the findings across different populations. This study aimed to address these gaps by analyzing a large, nationally representative cohort from the NHANES database.

## Materials and methods

### Study population

This investigation utilized data from the National Health and Nutrition Examination Survey (NHANES) (20), a large-scale nationwide assessment organized by the Centers for Disease Control and Prevention (CDC) to monitor the health and nutritional conditions of people living in the United States. Using a multistage random sample approach, data were gathered from 2005–2006, 2007–2008, 2009–2010, 2011–2012, and 2013–2014. Ethical approval for the study was granted by the Institutional Review Board of the National Center for Health Statistics (NCHS), and informed consent was obtained from all participants.

**Figure 1:**
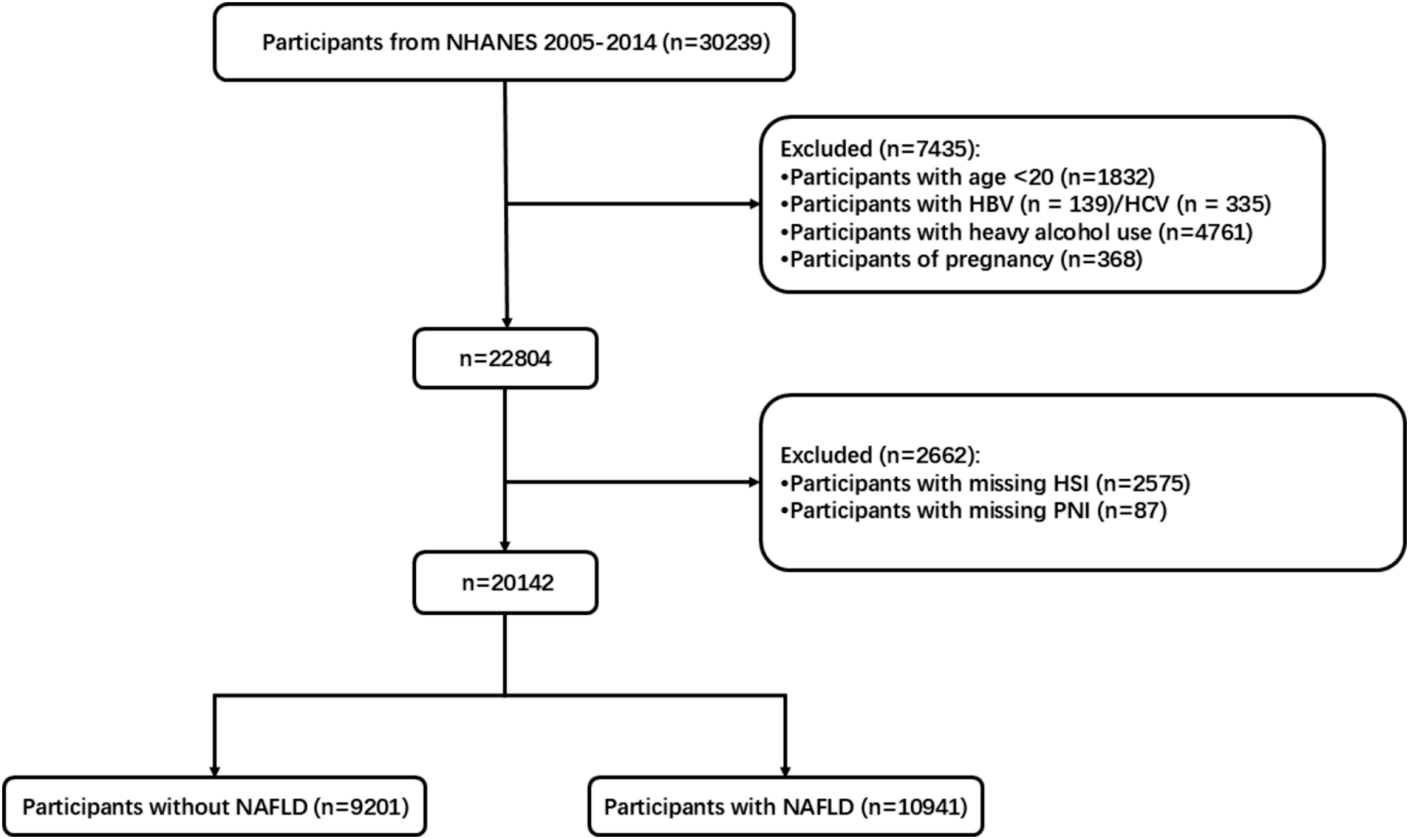
The flow of participant inclusion and exclusion in this study.

The data used in this analysis came from five NHANES cycles between 2005 and 2014. The assessment of hepatic steatosis through liver ultrasound, CT scans, or liver biopsy was lacking in most of the interview cycles. To diagnose NAFLD, the Hepatic Steatosis Index (HSI) was employed, calculated as HSI = 8 × (ALT/AST) + BMI, with an additional 2 points if the participant has diabetes and another 2 points if female. The HSI model demonstrated an AUC of 0.812, with thresholds set at 30 and 36. When values were either below 30 or above 36, the model showed a sensitivity of 93.1% for ruling out NAFLD and a specificity of 92.4% for identifying it (21). NAFLD was defined by an HSI > 36. The following conditions were used to exclude participants from the analysis: (1) individuals under 20 years of age (*n* = 1832); (2) those with hepatitis B (*n* = 139) or hepatitis C (*n* = 335); (3) heavy alcohol consumers (≥3 glasses for women or ≥ 4 glasses for males each day, *n* = 4,761); (4) pregnancy (*n* = 368); (5) those with missing HSI data (*n* = 2,575) (6) those with missing PNI data (*n* = 87) (Figure 1). Following the use of these exclusion criteria, 10,007 NAFLD subjects made up the final cohort. These individuals were included to investigate the connection between the PNI and both overall and cardiovascular disease mortality.

### Assessment of PNI

The blood count was completed by measuring hematological parameters using the automatic blood analyzer DxH 900. PNI was defined according to serum albumin and blood lymphocyte levels (22). Serum albumin levels were multiplied by five times the blood lymphocyte count to determine PNI.

### Covariates

For this analysis, baseline data were gathered from NHANES participants through standardized surveys, lab tests, and clinical assessments. The demographic characteristics included sex, age, race, education level, and marital status. The poverty-to-income ratio (PIR) was used to assess socioeconomic status. Clinical variables included BMI (23), smoking status, hypertension (defined either by self-reported diagnosis, ongoing antihypertensive medication use, or having an average blood pressure at or above 140 mmHg for systolic and 90 mmHg for diastolic readings), and diabetes (self-reported history, medication use, or meeting American Diabetes Association diagnostic criteria) (24). CVD history was determined based on self-reported diagnoses of angina, stroke, myocardial infarction, heart failure or coronary artery disease (25). Laboratory markers included serum creatinine (Cr), serum albumin (ALB), alanine aminotransferase (ALT), aspartate aminotransferase (AST), high-density lipoprotein cholesterol (HDL), total cholesterol (TC), triglycerides (TG), lymphocyte count (LYM), and hemoglobin A1c (HbA1c). The FIB-4 index, used to assess the likelihood of liver fibrosis, was calculated as (age multiplied by AST) divided by the product of platelet count and the square root of ALT. A score exceeding 2.67 was identified as a marker of significant or advanced fibrosis (26).

### Mortality assessment and follow-up

To estimate mortality, this study utilized data from the National Death Index (NDI), which was linked to the NHANES public-use mortality files spanning from 2005 to 2014. The follow-up period extended until December 31, 2019. The main focus of the study was all-cause mortality, while deaths attributable to specific diseases, including those from CVD conditions (coded I00-I09, I11, I13, I20-I51, I60-I69), were identified according to the 10th edition of the International Classification of Diseases (ICD-10).

### Statistical analyses

This study employed 14-year weights, stratification, and clustering, to account for the intricate, multistage sampling methodology of NHANES (20). The sampling weight formula used was: 14-year fasting subsample MEC weight = 2-year fasting subsample MEC weight/7. All statistical analyses were carried out with EmpowerStats 4.2 and R version 4.1.1. Weighted averages with 95% CI were used to report continuous data, whereas weighted proportions were used to represent categorical data. Differences in continuous variables were evaluated via weighted linear regression, while categorical variables were evaluated using weighted chi-square tests.

PNI values were classified into four categories, with the reference category being the first quartile. HR and 95% CI were calculated to compare the higher quartiles (second, third, and fourth) against the lowest quartile. PNI was also analyzed as a continuous variable. A survey-weighted Cox proportional hazard was employed to assess a link between PNI and mortality risks from all causes and CVD disease in NAFLD patients. To control for confounding variables, three distinct models were used: the first model was unadjusted, the second model accounted for sex, age, race, educational attainment, marital status, PIR, BMI, and smoking habits, and the third model included additional adjustments for advanced liver conditions, such as HIS, fibrosis severity, as well as hypertension, diabetes, and history of CVD events. Kaplan–Meier curves were built to depict the link between PNI levels and mortality outcomes, focusing on both total and cardiovascular-related deaths within the NAFLD cohort. To examine how PNI correlates with mortality in NAFLD patients in a non-linear manner, we utilized Cox regression analysis enhanced by cubic spline functions and smooth curve fitting via the penalized spline approach. To assess the threshold effect of PNI on mortality, we employed a two-piecewise linear regression model combined with a smoothing function. The threshold (or turning point) was identified through trial and error, where potential turning points within a predefined range were tested, and the point yielding the highest model likelihood was selected. Additionally, we performed a likelihood ratio test to compare the fit of the two-piecewise linear regression model with that of a simpler single-line model. We further explored the relationship between PNI and all-cause mortality by conducting subgroup analyses and grouping participants according to factors such as sex, age, education, marital status, race, BMI, family poverty income ratio, HIS, advanced fibrosis, hypertension, diabetes, and CVD. Interaction terms were also evaluated to identify potential modifying effects. For cardiovascular mortality, similar subgroup analyses were performed. Additionally, to assess how effectively PNI predicts survival over time, we conducted a time-dependent ROC curve analysis. To minimize possible bias in excluding missing data from the analysis, we used multiple interpolations to deal with missing values. All tests were evaluated using a statistical criterion of *p* < 0.05.

## Results

### Characteristics of study participants with or without NAFLD

This investigation examined the baseline profiles of American adults both with and without NAFLD, as shown in Table 1. It was observed that the group of NAFLD participants was a notably older average age than that of their without NAFLD counterparts (*p* = 0.004). Differences in race distribution were additionally discovered, with a higher prevalence of non-Hispanic Black and Mexican American participants in NAFLD (*p* < 0.001). NAFLD patients exhibited higher mean BMI, TG, TC, LYM, and HbA1c levels, along with lower HDL (all *p* < 0.001). Hypertension, diabetes, and CVD disease were more common among NAFLD patients (all *p* < 0.001), indicating a distinct metabolic risk profile. Additionally, significant differences in education level, marital status, and income were noted (all *p* < 0.001). Participants with NAFLD also had a poorer PNI than non-NAFLD individuals (*p* < 0.001), indicating a potentially poorer nutritional status in this group.

**Table 1:** The weighted clinical features of participants with and without NAFLD.

Variable	All (*n* = 20,142)	Non-NAFLD (*n* = 9,201)	NAFLD (*n* = 10,941)	*p*-value
Age (years)	49.59 (49.05, 50.12)	49.02 (48.32, 49.72)	50.09 (49.52, 50.67)	0.004
Sex (%)				0.356
Male	46.32 (45.62, 47.02)	46.70 (45.61, 47.80)	45.97 (44.96, 46.99)	
Female	53.68 (52.98, 54.38)	53.30 (52.20, 54.39)	54.03 (53.01, 55.04)	
Race (%)				<0.001
Mexican American	7.17 (5.94, 8.62)	5.28 (4.46, 6.24)	8.86 (7.22, 10.83)	
Non-Hispanic Black	10.98 (9.52, 12.63)	8.98 (7.77, 10.34)	12.78 (10.98, 14.82)	
Non-Hispanic White	69.99 (66.92, 72.90)	72.31 (69.53, 74.93)	67.91 (64.34, 71.28)	
Other	11.86 (10.54, 13.32)	13.43 (11.93, 15.09)	10.45 (9.07, 12.02)	
Education (%)				<0.001
Less than high school	6.07 (5.43, 6.78)	5.40 (4.76, 6.11)	6.67 (5.86, 7.58)	
High school	32.70 (31.07, 34.37)	30.25 (28.32, 32.25)	34.89 (33.20, 36.63)	
High school above	61.23 (59.30, 63.13)	64.35 (62.06, 66.58)	58.44 (56.50, 60.35)	
Marital status (%)				<0.001
Married	67.04 (65.79, 68.27)	65.11 (63.33, 66.86)	68.77 (67.53, 69.99)	
Separated	12.29 (11.70, 12.90)	11.21 (10.35, 12.14)	13.25 (12.47, 14.08)	
Never married	20.67 (19.49, 21.90)	23.68 (21.99, 25.45)	17.97 (16.87, 19.14)	
PIR (%)	3.04 (2.97, 3.12)	3.15 (3.06, 3.24)	2.95 (2.88, 3.03)	<0.001
BMI (kg/m^2^)	28.82 (28.66, 28.98)	23.90 (23.82, 23.98)	33.23 (33.05, 33.41)	<0.001
ALB (g/L)	42.63 (42.54, 42.73)	43.29 (43.17, 43.40)	42.05 (41.94, 42.15)	<0.001
ALT (U/L)	24.56 (24.31, 24.81)	20.32 (20.04, 20.60)	28.36 (27.97, 28.76)	<0.001
AST (U/L)	25.07 (24.86, 25.27)	24.61 (24.24, 24.98)	25.48 (25.21, 25.74)	<0.001
CR (mg/dL)	0.91 (0.90, 0.91)	0.91 (0.90, 0.92)	0.91 (0.90, 0.92)	0.832
HDL (mg/dL)	53.05 (52.65, 53.44)	58.21 (57.67, 58.75)	48.42 (48.05, 48.79)	<0.001
TG (mg/dL)	153.39 (150.87, 155.90)	123.61 (121.23, 125.99)	180.08 (176.39, 183.77)	<0.001
TC (mg/dL)	195.51 (194.61, 196.41)	193.27 (192.14, 194.41)	197.52 (196.45, 198.59)	<0.001
LYM (×10^9^/L)	2.10 (2.08, 2.12)	2.01 (1.98, 2.03)	2.19 (2.16, 2.21)	<0.001
HBA1C (%)	5.63 (5.61, 5.65)	5.42 (5.40, 5.43)	5.83 (5.80, 5.86)	<0.001
Smoking (%)				<0.001
Never	58.41 (57.11, 59.70)	58.28 (56.64, 59.91)	58.52 (57.06, 59.96)	
Former	25.34 (24.35, 26.34)	23.44 (22.35, 24.57)	27.03 (25.76, 28.34)	
Now	16.26 (15.34, 17.22)	18.28 (16.83, 19.81)	14.45 (13.54, 15.40)	
Hypertension (%)				<0.001
No	66.26 (65.11, 67.40)	75.63 (74.34, 76.87)	57.87 (56.46, 59.26)	
Yes	33.74 (32.60, 34.89)	24.37 (23.13, 25.66)	42.13 (40.74, 43.54)	
Diabetes (%)				<0.001
No	86.92 (86.18, 87.63)	95.00 (94.40, 95.54)	79.68 (78.54, 80.77)	
Yes	13.08 (12.37, 13.82)	5.00 (4.46, 5.60)	20.32 (19.23, 21.46)	
CVD (%)				<0.001
No	90.49 (89.86, 91.09)	91.48 (90.66, 92.24)	89.61 (88.83, 90.33)	
Yes	9.51 (8.91, 10.14)	8.52 (7.76, 9.34)	10.39 (9.67, 11.17)	
Advanced fibrosis (%)				<0.001
No	97.11 (96.79, 97.40)	96.15 (95.61, 96.62)	97.97 (97.66, 98.25)	
Yes	2.89 (2.60, 3.21)	3.85 (3.38, 4.39)	2.03 (1.75, 2.34)	
HSI	37.55 (37.37, 37.73)	31.03 (30.90, 31.16)	43.40 (43.21, 43.59)	<0.001
PNI	53.14 (53.01, 53.28)	53.32 (53.16, 53.48)	52.99 (52.82, 53.16)	<0.001

Data are expressed as weighted percentages (95% CI). PIR, poverty income ratio; ALB, Albumin; AST, aspartate aminotransferase; ALT, alanine aminotransferase; CR, creatinine; TC, total cholesterol; TG, triglyceride; LYM, lymphocyte; HDL, high-density lipoprotein; BMI, body mass index; HbA1c, glycosylated hemoglobin A1c; CVD, cardiovascular disease; HIS, hepatic steatosis index; PNI, prognostic nutritional index.

### Relationship between the PNI and NAFLD individuals

According to this research, lower PNI was linked to older age, female sex, a higher proportion of non-Hispanic Black individuals, lower ALB levels, and adverse metabolic profiles, including higher BMI and HbA1c (all *p* < 0.001) (Table 2). Additionally, lower PNI corresponded to a higher prevalence of diabetes, CVD disease, and advanced fibrosis. Conversely, participants in the higher PNI quartiles were younger, predominantly male, and had more favorable metabolic profiles, as well as a lower prevalence of diabetes, CVD disease, and advanced fibrosis (Table 2). These findings suggested that lower PNI was indicative of a more adverse metabolic status and a higher overall disease burden among this population.

**Table 2:** Baseline features based on weighted prognostic nutritional index quartiles.

Variable	PNI				*p* value
	Q1 < 49.000	Q2 49.000–52.000	Q3 52.500–55.000	Q4 > 55.50	
Age (years)	55.14 (54.27, 56.01)	51.95 (51.12, 52.77)	49.31 (48.48, 50.14)	45.53 (44.78, 46.28)	<0.001
Sex (%)					<0.001
Male	33.94 (31.34, 36.63)	41.99 (39.65, 44.36)	49.20 (46.77, 51.63)	55.29 (53.30, 57.26)	
Female	66.06 (63.37, 68.66)	58.01 (55.64, 60.35)	50.80 (48.37, 53.23)	44.71 (42.74, 46.70)	
Race (%)					<0.001
Mexican American	6.81 (5.31, 8.69)	7.95 (6.32, 9.96)	9.86 (7.98, 12.11)	10.27 (8.30, 12.64)	
Non-Hispanic Black	18.41 (16.01, 21.08)	13.11 (10.99, 15.56)	10.97 (9.20, 13.02)	10.03 (8.24, 12.16)	
Non-Hispanic White	67.54 (63.72, 71.14)	70.66 (66.76, 74.29)	68.28 (64.15, 72.14)	65.42 (61.20, 69.40)	
Other	7.24 (5.86, 8.91)	8.28 (6.93, 9.87)	10.89 (9.04, 13.08)	14.28 (12.12, 16.75)	
Education (%)					0.258
Less than high school	6.76 (5.74, 7.95)	6.05 (5.03, 7.26)	7.23 (6.14, 8.49)	6.66 (5.54, 8.00)	
High school	34.53 (31.94, 37.21)	34.44 (32.15, 36.81)	33.76 (31.39, 36.22)	36.54 (33.99, 39.18)	
High school above	58.71 (56.01, 61.36)	59.51 (56.86, 62.11)	59.01 (56.50, 61.48)	56.79 (53.88, 59.66)	
Marital status (%)					0.006
Married	69.99 (67.88, 72.03)	69.75 (68.03, 71.42)	69.21 (66.63, 71.68)	66.65 (64.42, 68.81)	
Separated	14.11 (12.92, 15.38)	13.68 (12.14, 15.39)	12.27 (10.88, 13.81)	13.12 (11.58, 14.83)	
Never married	15.90 (14.17, 17.79)	16.57 (14.92, 18.35)	18.52 (16.59, 20.63)	20.23 (18.34, 22.25)	
PIR (%)	2.87 (2.79, 2.95)	2.99 (2.89, 3.10)	3.05 (2.95, 3.15)	2.89 (2.78, 3.00)	0.004
BMI (kg/m^2^)	34.99 (34.63, 35.34)	33.43 (33.10, 33.76)	32.55 (32.29, 32.82)	32.38 (32.12, 32.64)	<0.001
ALB (g/L)	38.83 (38.71, 38.95)	41.32 (41.21, 41.43)	42.76 (42.66, 42.86)	44.37 (44.24, 44.49)	<0.001
ALT (U/L)	23.87 (22.95, 24.79)	26.81 (26.07, 27.55)	29.45 (28.49, 30.40)	32.01 (31.19, 32.82)	<0.001
AST (U/L)	24.08 (23.46, 24.70)	25.03 (24.49, 25.56)	25.59 (25.09, 26.10)	26.78 (26.33, 27.22)	<0.001
CR (mg/dL)	0.96 (0.92, 1.00)	0.90 (0.88, 0.91)	0.90 (0.89, 0.91)	0.89 (0.88, 0.90)	0.007
HDL (mg/dL)	51.56 (50.84, 52.29)	49.59 (48.92, 50.27)	48.11 (47.47, 48.74)	45.40 (44.85, 45.95)	<0.001
TG (mg/dL)	147.50 (140.87, 154.13)	168.92 (163.43, 174.40)	180.34 (173.91, 186.77)	213.02 (206.06, 219.98)	<0.001
TC (mg/dL)	189.70 (187.62, 191.79)	195.15 (193.23, 197.07)	199.38 (197.32, 201.44)	203.57 (201.58, 205.57)	<0.001
LYM (×10^9^/L)	1.57 (1.55, 1.60)	1.90 (1.88, 1.92)	2.19 (2.17, 2.21)	2.88 (2.82, 2.94)	<0.001
HBA1C (%)	5.96 (5.91, 6.01)	5.85 (5.80, 5.91)	5.78 (5.74, 5.83)	5.74 (5.69, 5.79)	<0.001
Smoking (%)					<0.001
Never	60.06 (57.37, 62.68)	61.05 (58.54, 63.51)	57.32 (54.54, 60.05)	56.24 (54.05, 58.40)	
Former	29.75 (27.21, 32.42)	27.53 (25.45, 29.70)	28.24 (25.71, 30.92)	23.60 (21.74, 25.56)	
Now	10.19 (8.91, 11.64)	11.42 (9.80, 13.27)	14.44 (12.65, 16.44)	20.17 (18.25, 22.23)	
Hypertension (%)					<0.001
No	50.51 (47.67, 53.35)	55.39 (53.17, 57.59)	61.01 (58.99, 63.00)	62.57 (60.02, 65.06)	
Yes	49.49 (46.65, 52.33)	44.61 (42.41, 46.83)	38.99 (37.00, 41.01)	37.43 (34.94, 39.98)	
Diabetes (%)					<0.001
No	73.00 (70.59, 75.28)	79.19 (77.07, 81.16)	80.82 (78.85, 82.64)	83.89 (82.16, 85.48)	
Yes	27.00 (24.72, 29.41)	20.81 (18.84, 22.93)	19.18 (17.36, 21.15)	16.11 (14.52, 17.84)	
CVD (%)					<0.001
No	83.08 (81.17, 84.83)	89.15 (87.44, 90.66)	91.26 (89.85, 92.49)	93.22 (92.02, 94.26)	
Yes	16.92 (15.17, 18.83)	10.85 (9.34, 12.56)	8.74 (7.51, 10.15)	6.78 (5.74, 7.98)	
Advanced fibrosis (%)					<0.001
No	95.22 (94.23, 96.05)	98.02 (97.33, 98.54)	98.90 (98.42, 99.24)	99.10 (98.58, 99.43)	
Yes	4.78 (3.95, 5.77)	1.98 (1.46, 2.67)	1.10 (0.76, 1.58)	0.90 (0.57, 1.42)	
HSI	44.63 (44.26, 45.01)	43.45 (43.08, 43.82)	42.93 (42.65, 43.21)	42.89 (42.63, 43.16)	<0.001

PIR, poverty income ratio; ALB, Albumin; AST, aspartate aminotransferase; ALT, alanine aminotransferase; CR, creatinine; TC, total cholesterol; TG, triglyceride; LYM, lymphocyte; HDL, high-density lipoprotein; BMI, body mass index; HbA1c, glycosylated hemoglobin A1c; CVD, cardiovascular disease; HIS, hepatic steatosis index; PNI, prognostic nutritional index.

### Associations between PNI and mortality among American adults with NAFLD

**Figure 2:**
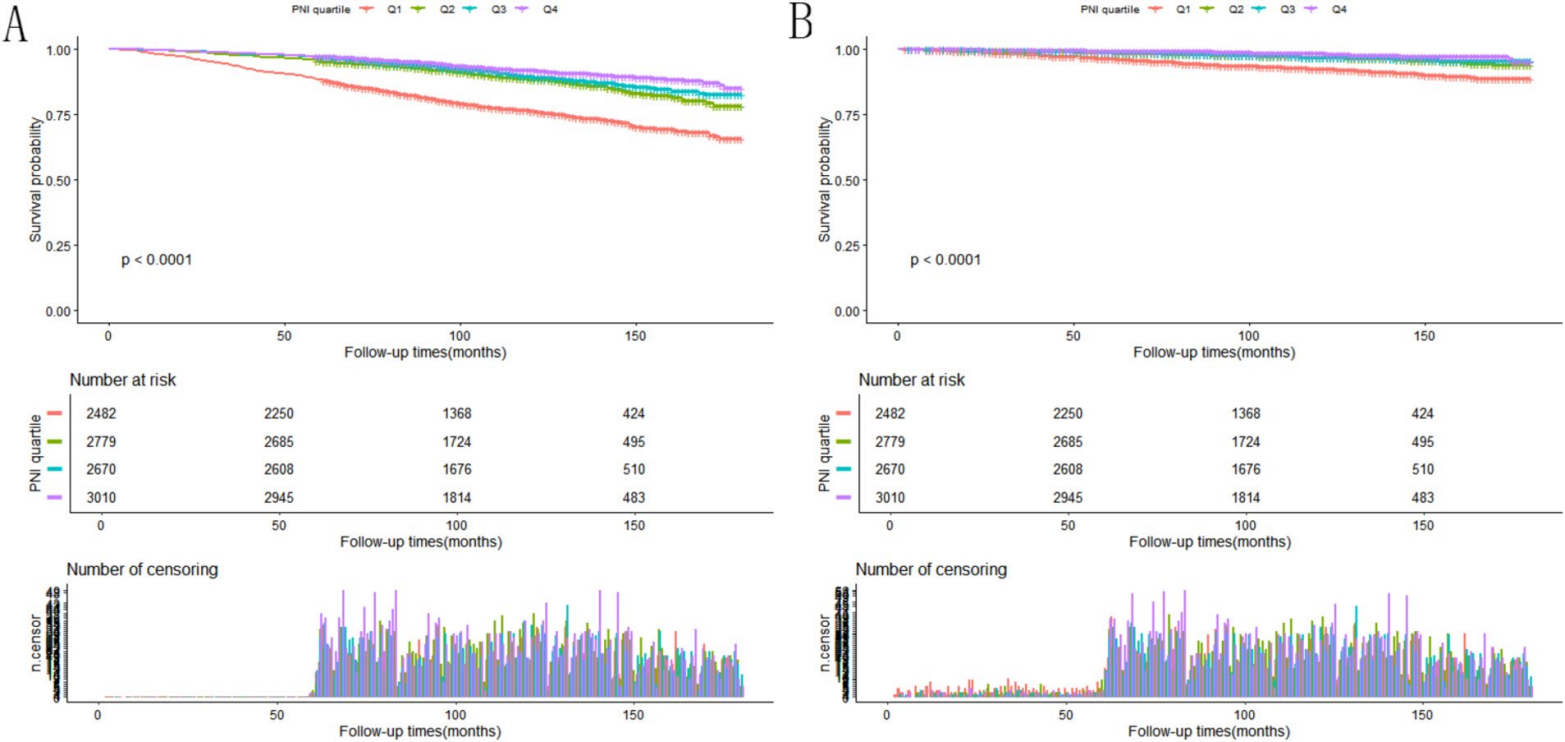
**(A)** Kaplan–Meier survival analysis depicting the rate of all-cause death among NAFLD patients stratified by PNI quartiles. **(B)** Survival curves derived from the Kaplan–Meier method showing cardiovascular mortality in NAFLD individuals, grouped by PNI quartile categories.

In this study, participants were observed over a median follow-up period lasting 119 months, with follow-up times spanning from 2 to 180 months. Throughout the study period, 1,438 participants died from all causes, and 392 deaths were due to CVD conditions among 10,941 participants with NAFLD. Figures 2A,B showed that, as illustrated by the Kaplan–Meier survival analysis, individuals with an elevated PNI experienced substantially reduced mortality from any cause and from cardiovascular disease specifically, in contrast to those with a lower PNI score (*p* < 0.0001). Higher PNI corresponded to a significantly reduced risk of mortality (Table 3), with each unit increase in PNI tied to a lower HR for all-cause mortality across each model (*p* < 0.05). Similarly, for CVD mortality, higher PNI values corresponded to lower HR (*p* < 0.001). The PNI quartiles consistently showed significant trends, indicating that a lower PNI independently indicated a greater death rate in this population.

**Table 3:** Relationship between PNI and the risk of all-cause as well as CVD mortality in individuals diagnosed with NAFLD.

	Model 1 HR (95% CI)	Model 2 HR (95% CI)	Model 3 HR (95% CI)
All-cause mortality			
PNI	0.90 (0.88, 0.93)	0.96 (0.93, 0.99)	0.96 (0.93, 0.99)
	<0.001	0.006	0.021
PNI quartile			
Q1	1 (Reference)	1 (Reference)	1 (Reference)
Q2	0.43 (0.36, 0.51)	0.53 (0.45, 0.63)	0.55 (0.46, 0.65)
Q3	0.38 (0.32, 0.45)	0.59 (0.51, 0.70)	0.62 (0.53, 0.72)
Q4	0.29 (0.25, 0.35)	0.59 (0.50, 0.69)	0.63 (0.54, 0.74)
	<0.001	<0.001	<0.001
*P* for Trend	<0.001	<0.001	<0.001
CVD mortality			
PNI	0.86 (0.83, 0.88)	0.91 (0.88, 0.95)	0.92 (0.88, 0.95)
	<0.001	<0.001	<0.001
PNI quartile			
Q1	1 (Reference)	1 (Reference)	1 (Reference)
Q2	0.39 (0.28, 0.54)	0.50 (0.35, 0.69)	0.51 (0.37, 0.71)
Q3	0.35 (0.26, 0.47)	0.58 (0.43, 0.76)	0.61 (0.46, 0.82)
Q4	0.17 (0.12, 0.25)	0.37 (0.25, 0.54)	0.41 (0.28, 0.60)
	<0.001	<0.001	<0.001
*P* for Trend	<0.001	<0.001	<0.001

Model 1: adjusted for none using appropriate sampling weights.

Model 2: adjusted for sex, age, education, marital status, race, BMI, and family poverty income ratio, using appropriate sampling weights.

Model 3: adjusted for sex, age, education, marital status, race, BMI, family poverty income ratio, HIS, advanced fibrosis, hypertension, diabetes, and CVD using appropriate sampling weights.

### Assessing the non-linear connection of PNI with mortality outcomes

**Figure 3:**
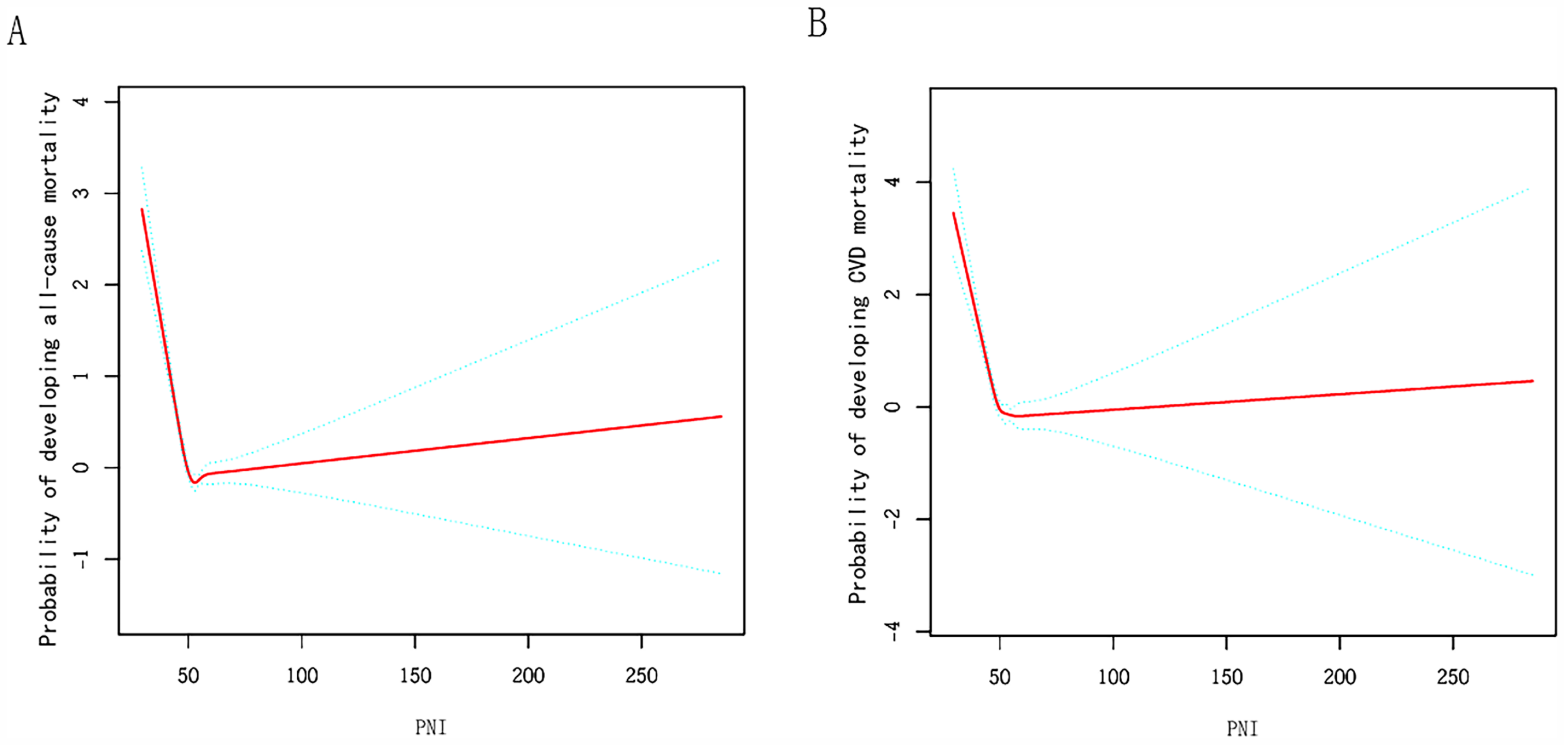
Relationships between PNI and the probability of **(A)** all-cause, **(B)** CVD mortality. Adjusted for sex, age, education, marital status, race, BMI, family poverty income ratio, HIS, advanced fibrosis, hypertension, diabetes, and CVD.

Our analysis, utilizing a Cox regression approach with penalized splines, uncovered a non-linear link between PNI scores and mortality likelihood (Figure 3). In Model I, a unit increase in PNI was linked to a marked decrease in all-cause (*p* < 0.001) and CVD mortality (*p* < 0.001). In Model II, a turning point was observed at PNI = 50.5. Below this point, the HR for all-cause mortality was 0.88 (*p* < 0.001), and above it, 1.00 (*p* = 0.258). Similar trends were observed for CVD mortality, suggesting a beneficial effect of higher PNI below the threshold (Table 4).

**Table 4:** Exploring the effect of PNI thresholds on the risk of all-cause and CVD mortality.

Models	Adjusted HR (95% CI)	*p*-value
All-cause mortality		
Model I		
One line slope	0.96 (0.94, 0.97)	<0.001
Model II		
Turning point (K)	50.5	
<50.5	0.88 (0.86, 0.89)	<0.001
>50.5	1.00 (1.00, 1.01)	0.258
HR between <50.5 and > 50.5	1.15 (1.12, 1.17)	<0.001
Logarithmic likelihood ratio test		<0.001
CVD mortality		
Model I		
One line slope	0.93 (0.91, 0.96)	<0.001
Model II		
Turning point (K)	50.5	
<50.5	0.85 (0.83, 0.88)	<0.001
>50.5	1.00 (0.99, 1.02)	0.722
HR between <50.5 and > 50.5	1.17 (1.13, 1.22)	<0.001
Logarithmic likelihood ratio test		<0.001

Adjusted for sex, age, education, marital status, race, BMI, family poverty income ratio, HIS, advanced fibrosis, hypertension, diabetes, and CVD.

### Subgroup analyses

**Figure 4:**
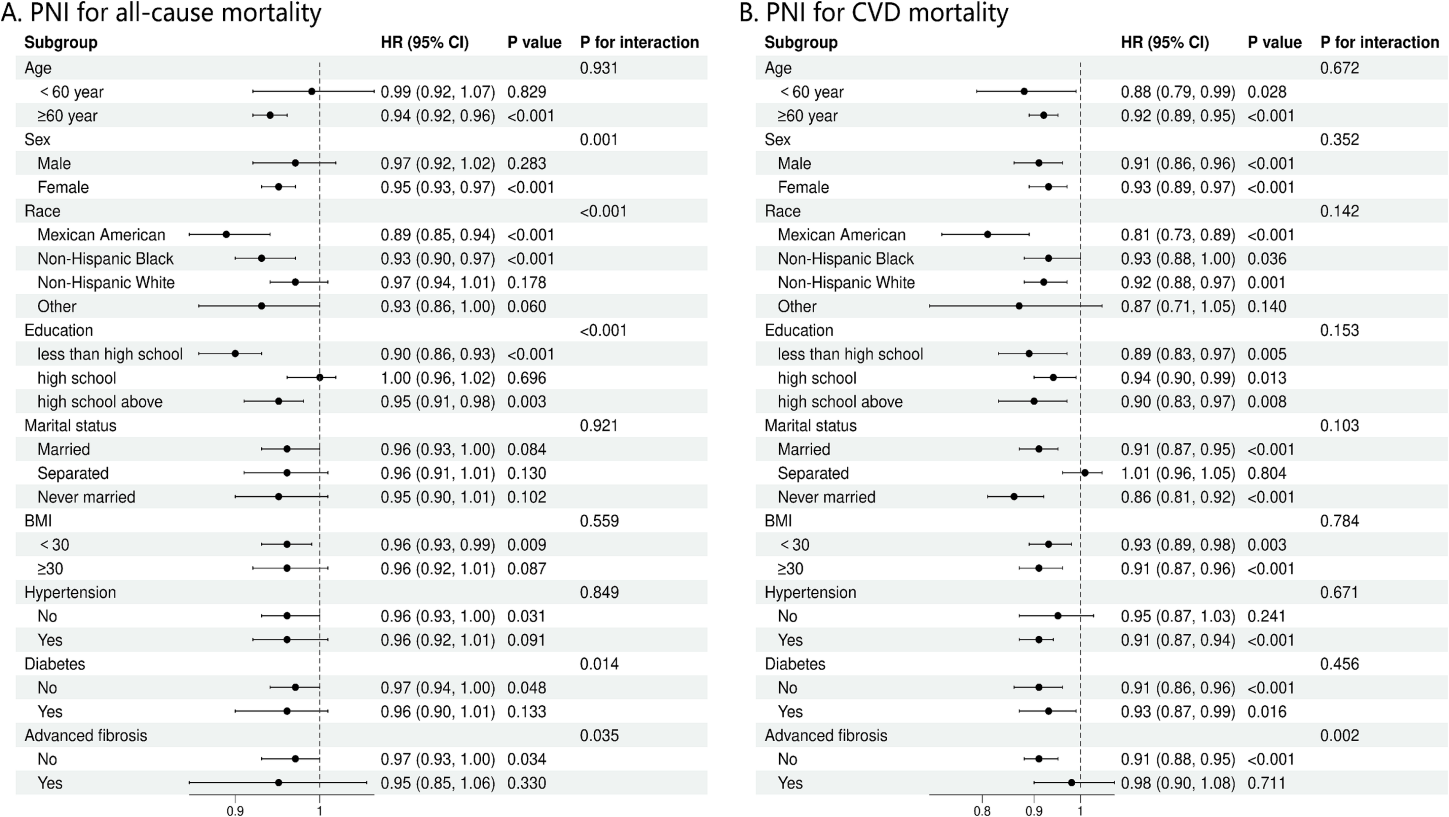
**(A)** Stratification analysis of PNI with mortality from all-cause using Cox regression analysis. **(B)** Stratification analysis of PNI with mortality from CVD using Cox regression analysis. CVD, cardiovascular disease; BMI, body mass index.

Our analysis revealed that the connection between PNI and the occurrence of all-cause mortality in NAFLD patients varied significantly across different demographic and clinical subgroups, after adjusting for multiple confounders (Figure 4A). Factors such as sex, race, education level, diabetes, and the stage of fibrosis were found to substantially influence this association (*P* for interaction = 0.001, <0.001, <0.001, 0.014, and 0.035, respectively). Subgroup study was conducted to assess the link between PNI levels and cardiovascular mortality, considering variations in age, sex, race, education, marital status, BMI, hypertension, diabetes, and the presence of advanced fibrosis (Figure 4B). The *P* for interaction indicates no significant interactions across most subgroups, except for advanced fibrosis (*p* = 0.002) (Figure 4B).

### Prognostic value of PNI

**Figure 5:**
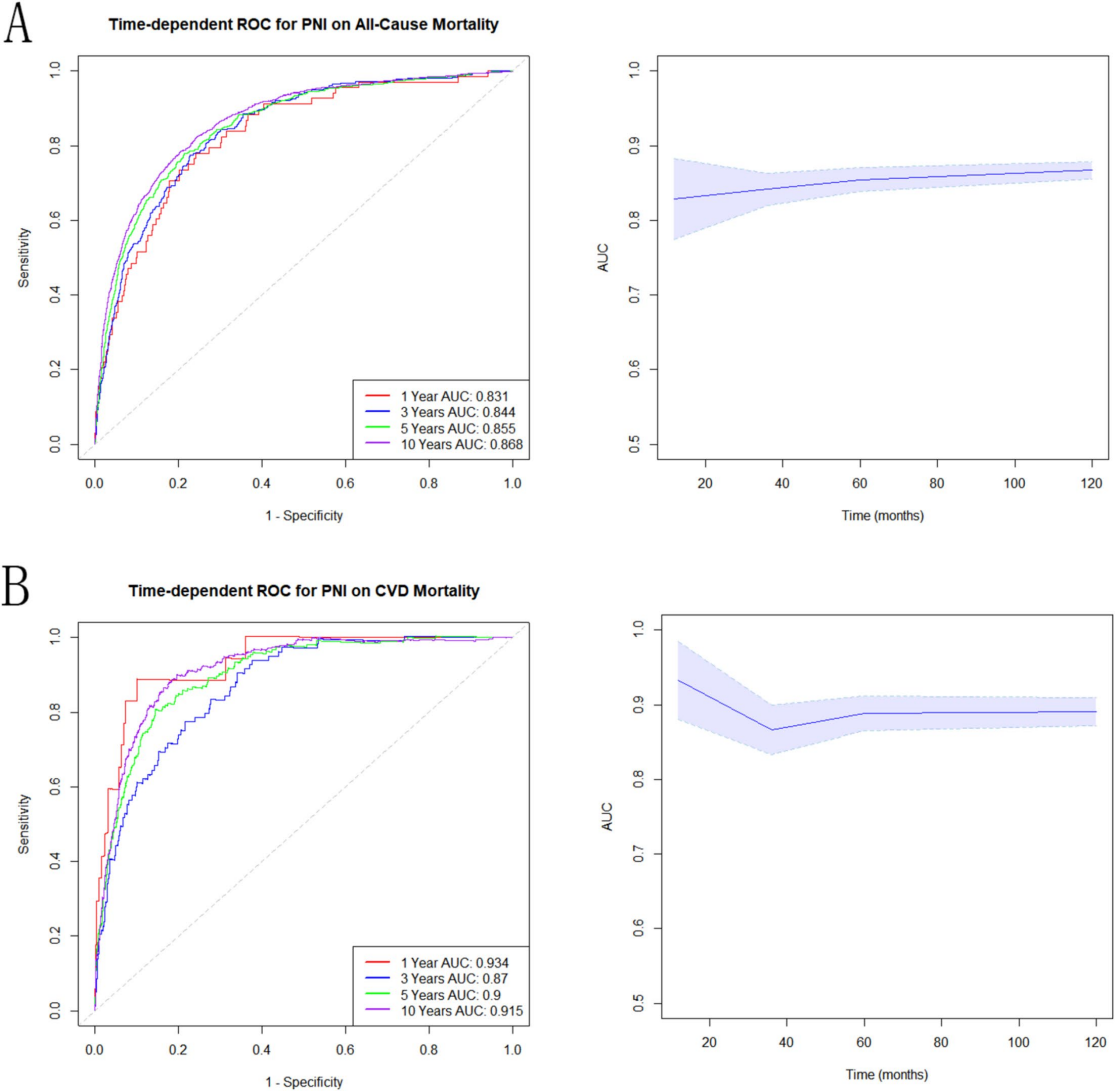
Associations of PNI with mortality in NAFLD. **(A)** Time-dependent ROC analysis illustrating the effectiveness of PNI in predicting all-cause mortality. **(B)** Time-dependent ROC evaluation of the role of PNI in predicting CVD mortality.

The connection between PNI and all-cause mortality (Figure 5A) and CVD (Figure 5B), was estimated using time-dependent receiver operating characteristics. According to the findings, the AUC of PNI was 0.831, 0.844, 0.855, and 0.868 for predicting all-cause death at 1, 3, 5, and 10 years (Figure 5A). For CVD mortality, PNI demonstrated AUC of 0.934, 0.87, 0.90, and 0.915 at 1, 3, 5, and 10 years (Figure 5B). According to these results, PNI exhibited a strong predictive potential for both short and long term all-cause and CVD mortality.

## Discussion

This study is, as far as we know, the inaugural analysis investigating how PNI impacts mortality among those diagnosed with NAFLD. The findings suggested that higher PNI levels were connected to reduced mortality and were a separate risk variable for little chance of surviving, and that this association persisted after controlling for a number of confounding factors. PNI was found to have a nonlinear connection with mortality. Higher PNI was linked to a reduced chance of death, especially below the PNI = 50.5 cutoff. Below this threshold, increased PNI significantly reduced the chance of death and showed a protective impact on death in NAFLD patients.

PNI serves as an effective indicator of immune nutritional status, calculated using total lymphocyte counts and serum albumin content. Serum albumin levels are a marker of liver function and nutritional status. Among NAFLD patients, decreased albumin can reflect impaired liver function, especially when the condition progresses to severe fibrosis or cirrhotic stages (27, 28). Hypoalbuminemia is frequently linked to an increased risk of mortality and complications with NAFLD because low albumin levels indicate systemic inflammation and poor liver synthesis (28, 29). Since NAFLD is a metabolic disease often co-occurring with factors like insulin resistance, excess weight, and other metabolic syndromes, maintaining adequate albumin levels is critical to support overall metabolic health and prevent disease exacerbations (30). The lymphocyte count is the second component of PNI and reflects immune function. In patients with NAFLD, chronic inflammation is the driving factor of disease progression, causing them to move from initial fatty liver changes to inflammatory non-alcoholic steatohepatitis and, in later stages, to liver cirrhosis (31). Reduced lymphocyte counts may indicate immunosuppression or dysfunction, which can exacerbate the progression of NAFLD by impacting the body’s capacity to manage inflammation and tissue repair (32). A lower lymphocyte count has been linked to a heightened mortality risk because immune capacity is essential in mitigating inflammation-related liver damage and preventing cardiovascular complications (33). Given the close link between inflammation, immune response, and metabolic dysfunction in NAFLD, PNI’s integration of albumin and lymphocyte levels provides a valuable tool for predicting prognosis. A higher PNI score indicates better immune nutritional status and is generally linked to reduced mortality among NAFLD patients. Conversely, lower PNI levels may suggest compromised immune function and nutritional deficiencies, potentially leading to elevated risks of complications in the liver, heart-related mortality, and death from any cause. In the context of NAFLD, assessing disease trajectory and anticipating patient prognosis may be effectively aided by using PNI as a meaningful evaluation tool.

PNI is a widely used, noninvasive, and cost-effective predictive method that has demonstrated good predictive power in research. In a report involving 393 patients with hypertrophic cardiomyopathy, higher PNI values (≥48.8) were associated with lower all-cause mortality (9.3% versus 33.1%) and cardiovascular mortality (7.1% versus 21.0%) (34). Even after adjusting for confounders, PNI showed a significant association with mortality as an independent predictor. Specifically, the risk ratios were 0.46 for deaths from any cause and 0.44 for deaths related to cardiovascular issues (34). In patients receiving hemodialysis, higher PNI values were significantly associated with lower mortality, superior to other markers such as serum albumin and lymphocyte count (35). Similarly, studies of overweight and obese cancer patients have confirmed that lower PNI values are linked to a heightened chance of death and poorer prognosis, further supporting the role of PNI in reflecting inflammation and nutritional status (15). These findings emphasize the utility of PNI as a key prognostic marker in diverse populations, particularly in assessing immune nutritional status and predicting mortality risk. In recent years, new nutritional indices like the Controlling Nutritional Status (CONUT) and the Geriatric Nutritional Risk Index (GNRI) have been developed and validated to assess the nutritional health of patients with NAFLD. Research showed that an inadequate nutritional state, as indicated by GNRI and CONUT, is positively associated with the risk of developing NAFLD, especially in individuals over the age of 50 (36). Another study highlighted that the Nutritional Risk Index (NRI) could potentially serve as a relevant biomarker linked to both NAFLD and liver fibrosis. Our study was different from other studies in terms of methodology and scope. As an innovative indicator, PNI has shown unique advantages in the prognosis assessment of patients with NAFLD, especially in the prediction of mortality. Kaplan–Meier analysis in our study demonstrated that elevated PNI levels were markedly linked to decreased rates of mortality. Multivariate Cox modeling indicated that mortality risks decreased with each unit increase in PNI, with a threshold effect observed at PNI = 50.5. Subgroup analyses showed consistent associations across most subgroups, with significant interactions between race, education, BMI, and advanced fibrosis. ROC analyses over time showed strong predictive power of the PNI, with consistently high AUC values for all-cause and CVD mortality at 1, 3, 5, and 10 years. PNI serves as an effective indicator for predicting long-term survival and risk of complications, identifying patients at high risk of NAFLD, and providing more targeted treatment.

The research offers significant advantages. By drawing on a large, nationally representative sample from NHANES, this study provided an extensive and reliable dataset for assessing the association between PNI and mortality among U.S. adults affected by NAFLD. The extended follow-up period enhances the robustness of the mortality results. In addition, PNI emphasizes its potential as a useful prognostic marker for mortality.

Despite the strengths of this study, certain constraints exist. First, the accuracy of HSI may be influenced by factors such as BMI, ALT, and AST levels, which can vary across populations, leading to potential variations in diagnostic performance. Second, HSI, being a non-invasive tool based on biochemical markers, lacked the diagnostic precision of more advanced techniques such as liver ultrasound, CT scans, or liver biopsy. Liver biopsy remains the gold standard for diagnosing NAFLD. Third, subgroup analyses were conducted without formal correction for multiple comparisons, which may increase the risk of spurious findings due to chance, as multiple tests raise the likelihood of detecting statistically significant associations occurring randomly. Fourth, the analysis relied on data sources, which might be prone to data missingness or measurement biases. Finally, due to the inability to establish a causal relationship between nutritional interventions targeting PNI and mortality rates associated with NAFLD, this study presented a degree of uncertainty in formulating clinical guidance. Determining if targeting PNI through specific interventions can effectively reduce or improve mortality rates associated with NAFLD remains a challenge. To further validate these findings, larger-scale studies involving more diverse NAFLD patient populations are needed.

## Conclusion

Through an analysis of 10,007 participants with NAFLD from 2005 to 2014 NHANES, our study highlights the long-term association between NAFLD and heightened risks of mortality. The results provide robust evidence in favor of incorporating PNI into routine clinical evaluations as a significant marker for mortality prediction.

## Data Availability

The datasets presented in this study can be found in online repositories. The names of the repository/repositories and accession number(s) can be found in the article/supplementary material.
